# Proper Blends of Biodegradable Polycaprolactone and Natural Rubber for 3D Printing

**DOI:** 10.3390/polym12102416

**Published:** 2020-10-20

**Authors:** Thossapit Wissamitanan, Charoenyutr Dechwayukul, Ekwipoo Kalkornsurapranee, Wiriya Thongruang

**Affiliations:** 1 Department of Mechanical Engineering, Faculty of Engineering, Prince of Songkla University, Hat Yai, Songkhla 90112, Thailand; thossapit.wis@gmail.com (T.W.); charoenyut.d@psu.ac.th (C.D.); 2 Department of Materials Science and Technology, Faculty of Science, Prince of Songkla University, Hat Yai, Songkhla 90112, Thailand; ekwipoo.k@psu.ac.th; 3 Center of Excellence in Metals and Materials Engineering, Faculty of Engineering, Prince of Songkla University, Hat Yai, Songkhla 90112, Thailand

**Keywords:** natural rubber, ENR-50, STR5L, PCL, 3D printing

## Abstract

Flexible thermoplastic elastomers (TPE) were prepared for fused deposition modeling (FDM) or 3D printing. These materials can be used for medical purposes such as disposable soft splints and other flexible devices. Blends of 50% epoxidized natural rubber (ENR-50) and block rubber (Standard Thai Rubber 5L (STR5L)) with polycaprolactone (PCL) were produced and compared. The purpose of this study was to investigate the properties of natural rubber (NR) and PCL in simple blends with PCL contents of 40%, 50%, and 60% by weight (except at 75% for morphology study) in the base mixture (NR/PCL). The significant flow factors for FDM materials, such as melting temperature (T_m_) and melt flow rate (MFR), were observed by differential scanning calorimetry (DSC) and via the melt flow index (MFI). In addition, the following mechanical properties were also determined: tensile strength, compression set, and hardness. The results from DSC showed that the melting temperature changed slightly (1–2 °C) with amount of PCL used, and there was a suspicious point in the 50/50 blends with both types of rubber. The lowest melting enthalpy of both blends was found at the 50/50 blended composition. The MFI results showed that PCL significantly affected the melt flow rate of both blends. The ENR-50/PCL blend flowed better than the STR5L/PCL blend. The conclusion was that this was due to the morphology of its phase structure having better uniformity than that of the STR5L/PCL blend. In compression set testing or measuring shape recovery, rubber directly influenced the recovery in all blends. The ENR-50/PCL blend had less recovery than the STR5L/PCL blend, probably due to the functional effects of epoxide groups and polarity mismatch. The hard phase PCL significantly affected the hardness of samples but improved shape recovery of the material. The ENR-50/PCL blend had better tensile properties than the STR5L/PCL blend. The elongation at break of both blends improved with a high rubber content. Hence, the ENR-50/PCL blend was superior to STR5L/PCL for printing purposes due to its better miscibility, uniformity, and flow, which are the keys to success for optimizing the fused deposition modeling conditions as well as the overall mechanical properties of products. Most blends in this study were only slightly different, but the 50/50 blend of ENR-50/PCL seemed to be near optimal for 3D printing.

## 1. Introduction

Rapid prototyping is an interesting innovation of this century, for industrial as well as for personal uses. The well-known rapid prototyping process for plastics is fused deposition modeling (FDM), also called 3D printing. 3D printers are used widely for both modeling and product forming, mainly due to their economy and ease of use [1]. They have been applied in various fields, for instance, medical [2,3], engineering [4], and military. The engineering applications include modeling and forming of complicated or replacement parts. The quality of 3D-printed products depends mainly on materials [5,6,7] and printing conditions [8,9]. The bulk materials used for 3D printing are thermoplastics, such as acrylonitrile butadiene styrene (ABS), polyethylene terephthalate (PET)*,* and polylactic acid (PLA). However, printing with elastic materials is still very limited except for thermoplastic polyurethane (TPU), a resilient plastic that is often used. Novel PLA and ABS filaments are the most commonly used FDM materials. In addition, they have been used as core materials [5,10,11,12] and also composites [13]. Their good properties for FDM are low melting temperature, good flow, and rather good mechanical properties. With these properties, the biodegradable PLA and polycaprolactone (PCL) plastics were accepted and became more popular. Compared to PLA, PCL is more flexible and has a lower glass transition temperature (T_g_) and melting temperature (T_m_). Natural rubber (NR) blended with thermoplastics was widely studied to improve properties such as strength, toughness, and hardness [14,15]. In addition, epoxidized natural rubber (ENR) mixed with PCL was studied [16,17], and it was found that PCL improved tensile strength [18] and hardness of NR [19]. PCL and ENR were found to be incompatible because of their polarity and nonpolarity [16]. Moreover, relevant studies reported that ENR was more miscible with plastics than the standard type and unmodified NR [14]. Sulfur and peroxide agents (dicumyl peroxide (DCP)) were used to cross-link NR and PCL blends [19,20]. The simple blend (i.e., without crosslinking) of NR/PCL was also studied and compared, and it was found that mechanical properties were slightly better with a vulcanized system than with the simple blend. However, the vulcanized system made the blends flow poorly [21,22].

The aim of this study was to form and investigate flexible NR/PCL blends for FDM use. Two types of NR (50% epoxidized natural rubber (ENR-50) and Standard Thai Rubber 5L (STR5L)) were chosen to blend with PCL in proportions to be determined by testing. Surface morphology was used to confirm and explain the mechanical–morphological relationships and miscibility of the blends. Two significant criteria, the melting temperature and the melt flow rate, were measured to optimize printing conditions. The following mechanical properties were also tested to determine suitable blend proportions: compression set, hardness, and tensile strength.

## 2. Materials and Methods

STR5L is a commercial natural rubber block, the acronym standing for “Standard Thai Rubber”. ENR-50 is epoxidized natural rubber with an epoxide level of 50 mole percent. Both 50% epoxidized natural rubber (ENR-50) and commercial natural rubber (STR5L) were chosen for this study. ENR-50 was obtained from Muang Mai Guthrie Public Co., Ltd. (Nakorn Sri Thammarat, Thailand), and STR5L was from Chana Latex Co., Ltd. (Songkhla, Thailand). The polycaprolactone (CAPA-6500) used came as 2- to 3-mm pellets. Other ingredients were an activator (zinc oxide (ZnO)) and an antioxidant agent (Wingstay L) obtained from Kij Paiboon Chemical Co., Ltd. (Bangkok, Thailand). Paraffinic oil (PO, grade A No.15) was purchased from GSP Products Co., Ltd. (Bangkok, Thailand). The rubber and PCL were blended in the desired weight ratio (*w*/*w*) and other ingredients were mixed in proportions given as parts per hundred of rubber (phr). The compound formulations used and sample code labels are given in Table 1.

### 2.1. Compounding Preparation

An internal mixer MX300 (Charoen Tut Co., Ltd., Samutprakarn, Thailand) was used for mixing. The mixer was operated at a constant rotor speed of 60 rpm and an initial chamber temperature of 40 °C. For mixing, ENR-50 or STR5L was loaded and warmed for 5 min. After that, zinc oxide and Wingstay L were added every 2 min. Lastly, paraffinic oil was continuously poured in for 5 min. A compound was taken out of the chamber for cooling at room temperature. Finally, PCL was melted at 100 °C for 5 min in the previous mixer before adding ENR-50 or STR5L compound and mixing with PCL for 5 min before unloading. All specimens of both simple blends were prepared for testing without vulcanization of the rubber. The mixing steps and timing are shown in Table 2.

### 2.2. Surface Morphology

Surface morphology was imaged with a scanning electron microscope (SEM), Quanta 400 (FEI, Hillsboro, OR, USA). Samples were prepared to 2-mm thickness and broken in liquid nitrogen. The rubber phase was stained with osmium for 12 h to be able to distinguish the phases. The SEM micrographs were taken with the electron backscattered diffraction (EBSD) technique at 1000×, 2000×, and 4000× magnifications. To observe the influences of PCL clearly, samples with 25/75, 50/50, and 75/25 ratios of NR/PCL were imaged for comparison. Rubber appears as light and PCL as dark phases in the images shown.

### 2.3. Thermal Property Testing

Differential scanning calorimetry (DSC 8500), (PerkinElmer, Waltham, MA, USA) was used in this study to observe the transition temperatures (T_g_ and T_m_) and heat of fusion of the blends (∆H). The sample was cooled with liquid nitrogen to −150 °C and heated to 180 °C at 5 °C/min. Data collected were used to assess FDM forming temperature.

### 2.4. Melt Flow Index (MFI) Testing

Melt flow rate (MFR) of the polymer blend was tested according to the ASTM D1238-13 (Method A) standard, using an MFR machine (Charoen Tut Co., Ltd., Samutprakarn, Thailand). The PCL and all blends were tested at 125 °C with a 2.16 kg load. MFR data are given as averages of three replicates, within 10 min, of the melted sample weight.

### 2.5. Compression Set Testing

The compression set is that percentage of deformation that was not recovered. The testing was according to ASTM D395 (Method B) standard using the compression set tester no.171 (Yasuda Seiki Seisakusho, Ltd., Hyogo, Japan). The thickness and the diameter of circular specimens were 12.5 ± 0.5 mm and 29 ± 0.5 mm, respectively. A sample was compressed to 25% of its original thickness. The samples at a specified temperature (22 °C) were placed in the testing device for 22 h. Based on ASTM D395, the oven temperature was set at 70 °C. At this temperature, however, DSC revealed that the blends were already melted (melting points at 48–51 °C). Therefore, room temperature was used for testing in this study. After pressing for 22 h at 22 °C, the sample was left for 30 min, and then its thickness was measured. Three samples of each type were tested. The recovery was calculated as follows:

(1) $$Compression\;set\;\left(\%\right)=\left[\left(T_{1}-\;T_{2}\right)\;/\;\left(T_{1}\;-\;T_{S}\right)\right]\;\times \;100$$

where *T*_1_ is the original thickness of the specimen, *T*_2_ is the final thickness of the specimen, and *T_S_* is the thickness of the space bars.

### 2.6. Hardness Testing

The hardness of rubber was tested according to ASTM D2240 by the Shore A method using Digitest II (Bareiss Prüfgerätebau GmbH, Oberdischingen, Germany) at room temperature (23.2 °C). Five locations on each sample, from a total of three samples of a blend, were subjected to this hardness test. The hardness tester worked automatically and gave readings within 3 s.

### 2.7. Tensile Testing

Tensile testing was performed according to ASTM D412 (die C) using a Zwick/Roell Z010 (Zwick Roell, Ulm, Germany). The 2-mm-thick sheet sample was formed at 100 °C by a hydraulic press. The specimen was cut with die C in a pneumatic cutter. Testing was conducted with a 100 N load cell at the rate of 500 mm/min. Five test pieces of each blend were tested, and averages were reported of maximum force, maximum stress, and elongation at break.

## 3. Results and Discussion

### 3.1. Surface Morphology

**Figure 1:**
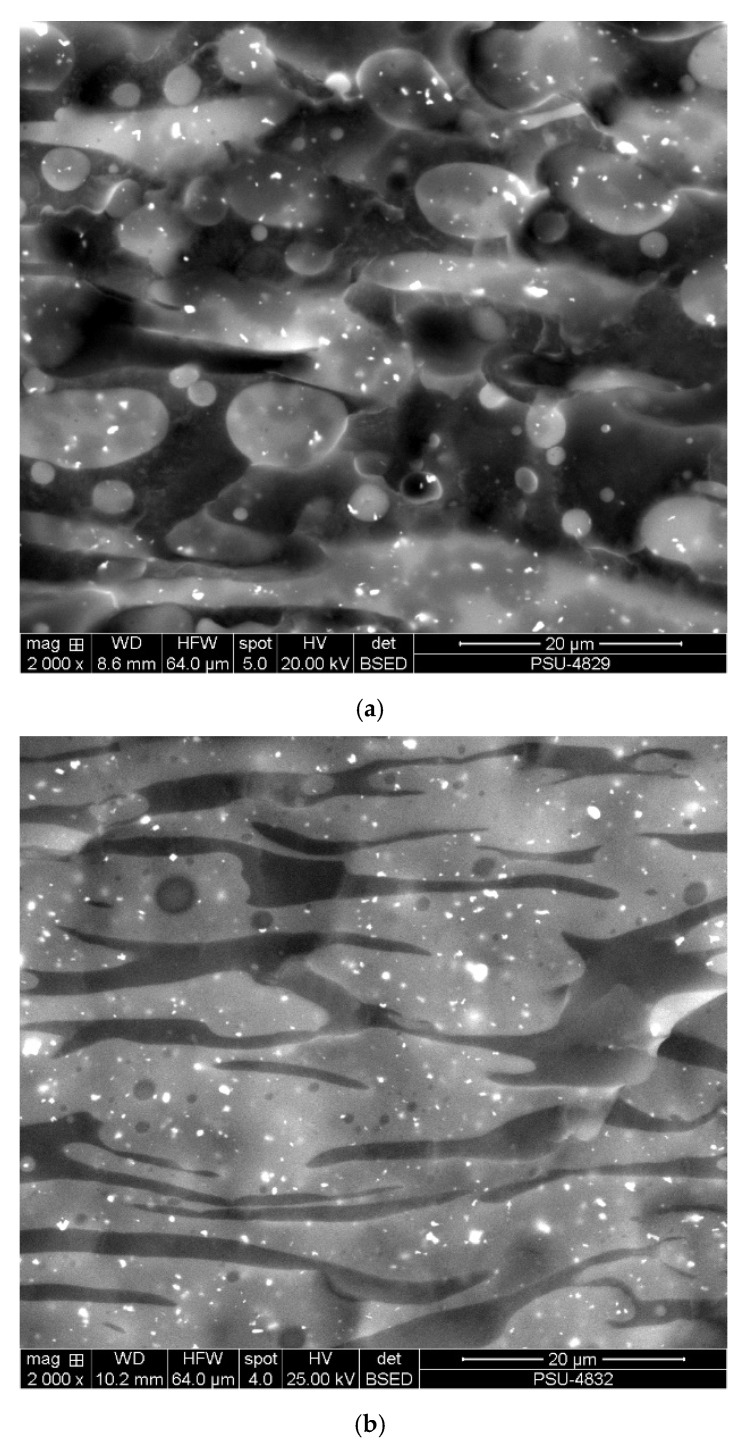

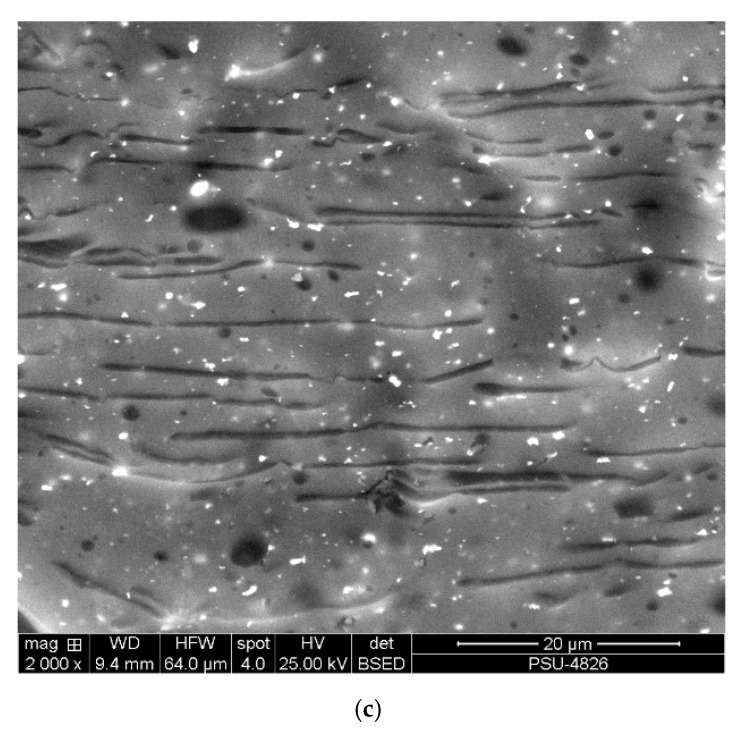
Fractured surface micrographs of Standard Thai Rubber 5L (STR5L)/polycaprolactone (PCL) blends: (**a**) STR5L/PCL 25/75, (**b**) STR5L/PCL 50/50, and (**c**) STR5L/PCL 75/25.

**Figure 2:**
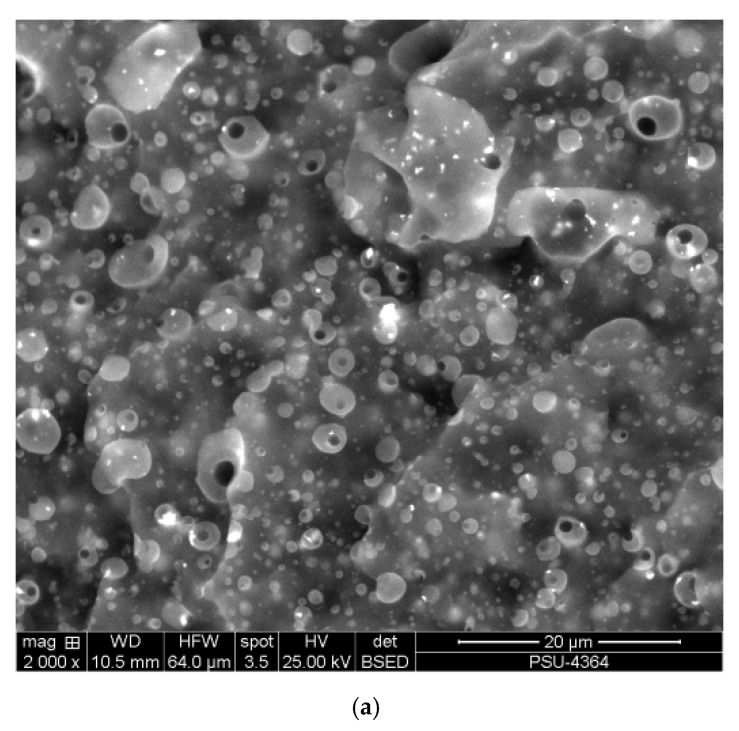

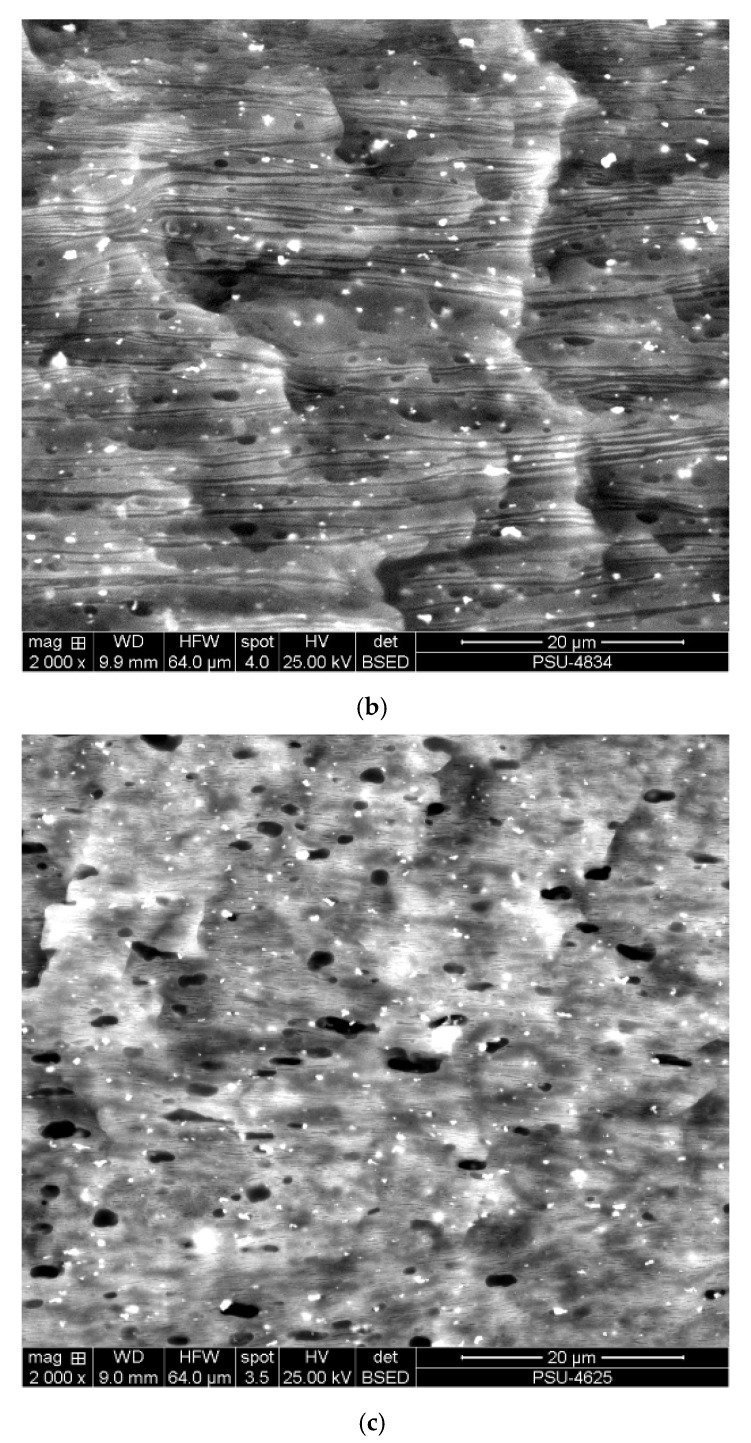
Fractured surface micrographs of 50% epoxidized natural rubber (ENR-50)/PCL blends: (**a**) ENR-50/PCL 25/75, (**b**) ENR-50/PCL 50/50, and (**c**) ENR-50/PCL 75/25.

Figure 1 and Figure 2 show morphological evidences from fractured surfaces of STR5L/PCL and ENR-50/PCL blends, respectively. Three compositions of the two types of rubber blended with PCL in ratios 25/75, 50/50, and 75/25 were studied. The rubber phase was stained with osmium in this study and is light colored in the images. The bright continuous phase is the rubber phase with areal fraction, consistent with the rubber content. Platelet and droplet domains were found with high contents of PCL. Morphologies of STR5L and ENR-50 blends are quite different and easy to distinguish. Weak interfacial interaction between STR5L and PCL was clearly shown from smooth surfaces and the separation of both phases. This was due to the polarity difference of both phases, similar to the previous study of the NR/PLA blend [14]. Hence, the clear and smooth fractured surface confirmed that the interaction and bonding between rubber and plastic was weak. For the ENR-50/PCL blend, micrographs revealed separations of the co-continuous phase with rough surfaces and finer grained structure than in the STR5L/PCL simple blend. Phase separation and weak bonding might be caused by polar PCL immiscible with the nonpolar part of ENR [16]. At 75% *w*/*w* of either rubber in the blend, large continuous phases were found and PCL was dispersed quite well in both rubber types, as shown in Figure 1c and Figure 2c, similar to the previous work [18]. It was concluded that ENR-50 mixed with PCL better than STR5L, as corroborated by the rather uniform distribution of phases in the ENR-50/PCL blends.

### 3.2. Melting Properties

Significant thermal characteristics for FDM are melting temperature and melt flow rate. They affect the ability to form the desired products with an FDM machine. The DSC results are shown in Table 3. The results revealed dual glass transition temperatures, primarily indicating phase separation of the blends. As found in this work, T_g_ of PCL in both blends tended to shift to a higher temperature, which might be caused by strong intermolecular interaction or bonding. However, to confirm this explanation, further studies of morphology, thermal analysis, and dynamic thermomechanical properties need to be co-analyzed. Compared to pure PCL, the melting temperature of PCL changed slightly by about 1–2 °C on adding STR5L or ENR-50, as in prior studies [14,22]. This is not expected to affect the suitability of PCL for FDM use. The melting temperatures of the ENR-50/PCL blends were higher than those of the STR5L/PCL blends, but insignificantly so. The ENR-50/PCL blends were superior in thermal stability when compared to the STR5L/PCL blends due to a higher crystallinity of PCL in the ENR-50/PCL than in the STR5L/PCL blend, according to the heat of fusion data. In addition, the migration of small molecules to the surface of STR5L can lead to chain scission of PCL, resulting in low thermal stability, as previously described [14,19,21]. Having STR5L or ENR-50 in the blend reduced the heat of fusion of PCL significantly, and the epoxide groups in ENR-50 improved thermal stability more than STR5L in the blends, as also found in a previous study [22]. It was also found from this work that the NR/PCL at 50/50 ratio gave the lowest melting temperature and the lowest heat of fusion as well.

**Figure 3:**
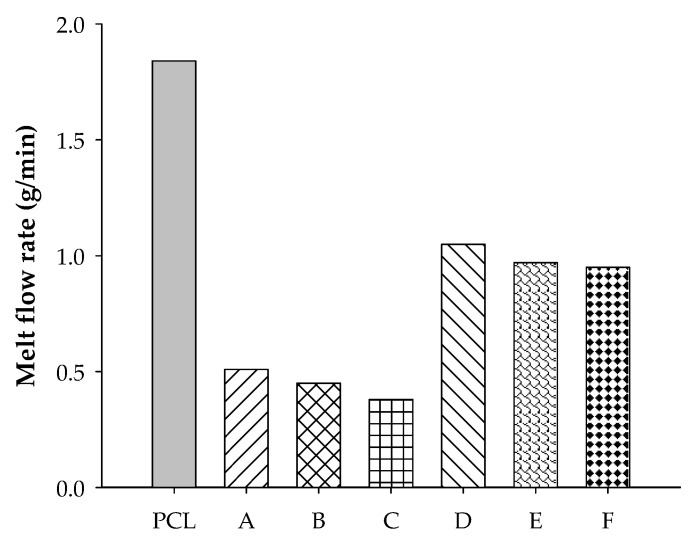
Melt flow index of PCL, A = STR5L/PCL at 40/60, B = STR5L/PCL at 50/50, C = STR5L/PCL at 60/40, D = ENR-50/PCL at 40/60, E = ENR-50/PCL at 50/50, and F = ENR-50/PCL at 60/40.

The PCL content in the blend positively affected the flowability, as shown in Figure 3. The results indicate that there were stronger interactions between ENR-50 and PCL than between STR5L and PCL, as described in the blended morphology section. Therefore, this resulted in a better flow of the ENR-50/PCL blend. It was found that both rubbers obstructed the flow of the blends, and ENR-50 showed a better compatibility with PCL than STR5L. From the melting properties, it is quite certain that the new blends of STR5L/PCL and ENR-50/PCL have the potential for use in FDM.

### 3.3. Compression Set

**Figure 4:**
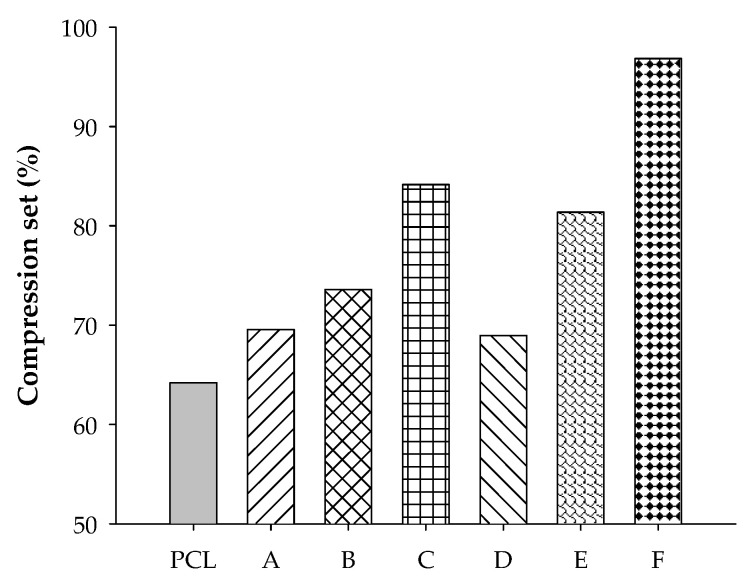
Compression set of PCL, A = STR5L/PCL at 40/60, B = STR5L/PCL at 50/50, C = STR5L/PCL at 60/40, D = ENR-50/PCL at 40/60, E = ENR-50/PCL at 50/50, and F = ENR-50/PCL at 60/40.

The compression set is a measure of shape recovery from a compressed state. A high compression set value indicates low recovery of materials. In this study, the compression set increased with content of rubber (STR5L or ENR-50), as shown in Figure 4. The compression set trends of PCL blends with ENR-50 and STR5L were similar. Comparing the results, it can be seen that PCL had a large beneficial impact on the recovery of shape. In general, materials with a high rubber content should have a high shape recovery, but this may not happen with blends having unvulcanized rubber that can flow or deform over a period of time. The epoxide groups in unvulcanized ENR-50 might cause poor shape recovery of the blend samples due to stronger chemical interactions between the epoxide groups in ENR-50 and the polar groups in PCL that might contribute to the loss of recovery and elasticity of PCL and ENR. This can be confirmed by the micrographs of the ENR-50/PCL blend, which revealed phase separation with a finer area of co-continuous structure.

### 3.4. Hardness

**Figure 5:**
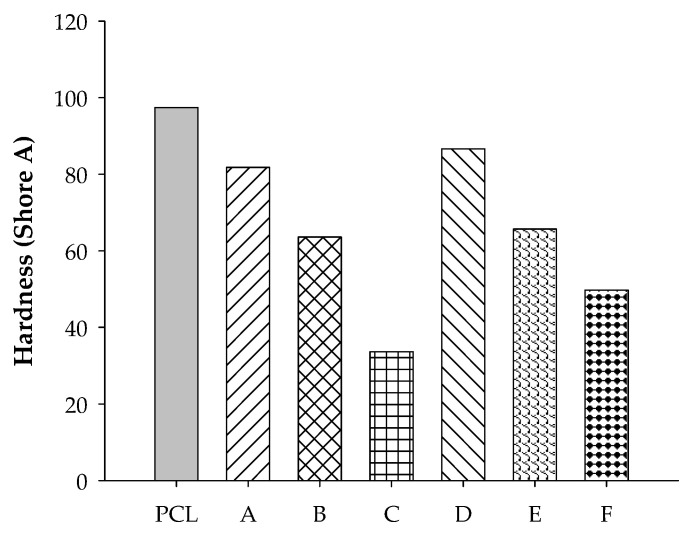
Hardness of PCL, A = STR5L/PCL at 40/60, B = STR5L/PCL at 50/50, C = STR5L/PCL at 60/40, D = ENR-50/PCL at 40/60, E = ENR-50/PCL at 50/50, and F = ENR-50/PCL at 60/40.

As expected, hardness decreased with rubber content in PCL blends since rubber is softer than PCL. Comparing the two rubber blends in Figure 5, it can be seen that the blends containing ENR-50 were slightly harder than those with STR5L. There was evidence confirming that a high level of the polar-epoxied group in ENR induced more chemical interaction and compatibilization between molecular segments of the blend, leading to the increase of hardness [15]. Both blends at 50/50 *w*/*w* had hardness in the range 63.6–65.7 Shore A, which corresponds to medium to soft rubber.

### 3.5. Tensile Testing

**Figure 6:**
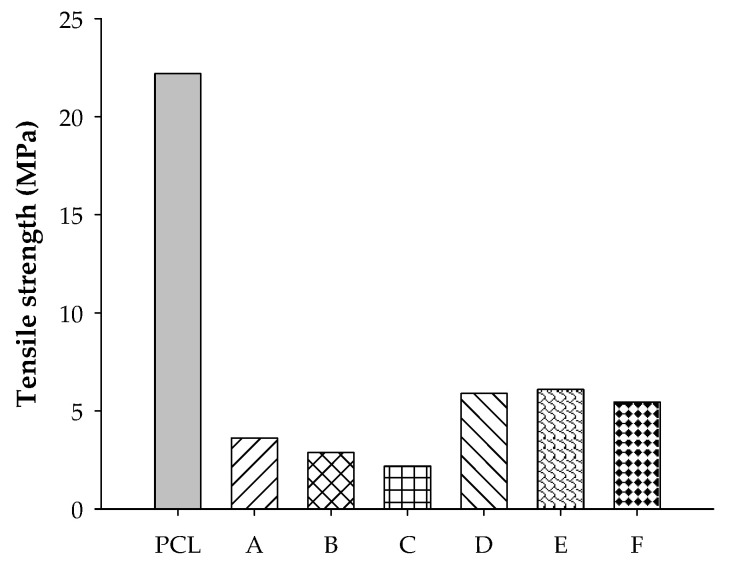
Tensile strength of PCL, A = STR5L/PCL at 40/60, B = STR5L/PCL at 50/50, C = STR5L/PCL at 60/40, ENR-50/PCL at 40/60, E = ENR-50/PCL at 50/50, and F = ENR-50/PCL at 60/40.

**Figure 7:**
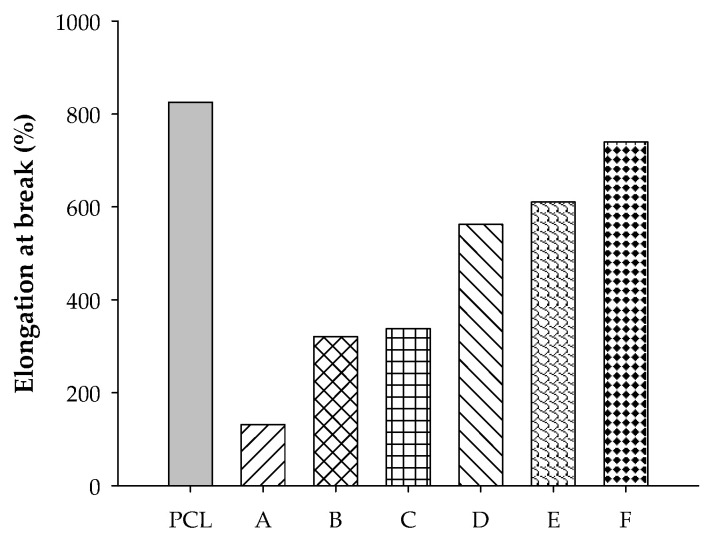
Elongation at break of PCL, A = STR5L/PCL at 40/60, B = STR5L/PCL at 50/50, C = STR5L/PCL at 60/40, D = ENR-50/PCL at 40/60, E = ENR-50/PCL at 50/50, and F = ENR-50/PCL at 60/40.

Tensile strength and elongation at break are shown in Figure 6 and Figure 7, respectively. The tensile strengths of both blends are much lower than that of the neat PCL. High rubber content tended to produce inferior strength of the samples. It can be seen from Figure 6 that ENR-50 blends can withstand loading better than STR5L blends, and this agrees well with previous work [23]. This was evidently supported by the results from SEM micrographs showing that there was a serious lack of the continuous rigid phase of PCL with incorporation of soft rubber. Simple blended samples of ENR-50 showed better strength than those of the STR5L due to ENR-50 being more miscible and compatible with PCL than STR5L, creating high continuity of the rigid PCL phase and leading to a high strength of the samples. In contrast to strength, the elongation at break increased with rubber content in the blends, as shown in Figure 7. The ENR-50/PCL blend was found to have much higher elongation at break (about 1.5–2.5 times) than that of the STR5L/PCL blend in the range of this study. The high interfacial bonding of the continuous phase plays a key role in the effect on the ultimate stretch of the samples. Compared to the elongation at break of both raw rubbers reported in the range of 1000–1100%, it can be concluded from the elongation results in this work that incorporating rubber in PCL (or vice versa) will cause each component and the blended sample as a whole to lose its ability to stretch due to the loss of its own phase continuity. However, the vulcanization of rubber was expected to improve strength and stretching ability of the blended samples and will be further studied.

### 3.6. Preliminary Filament Extrusion

**Figure 8:**
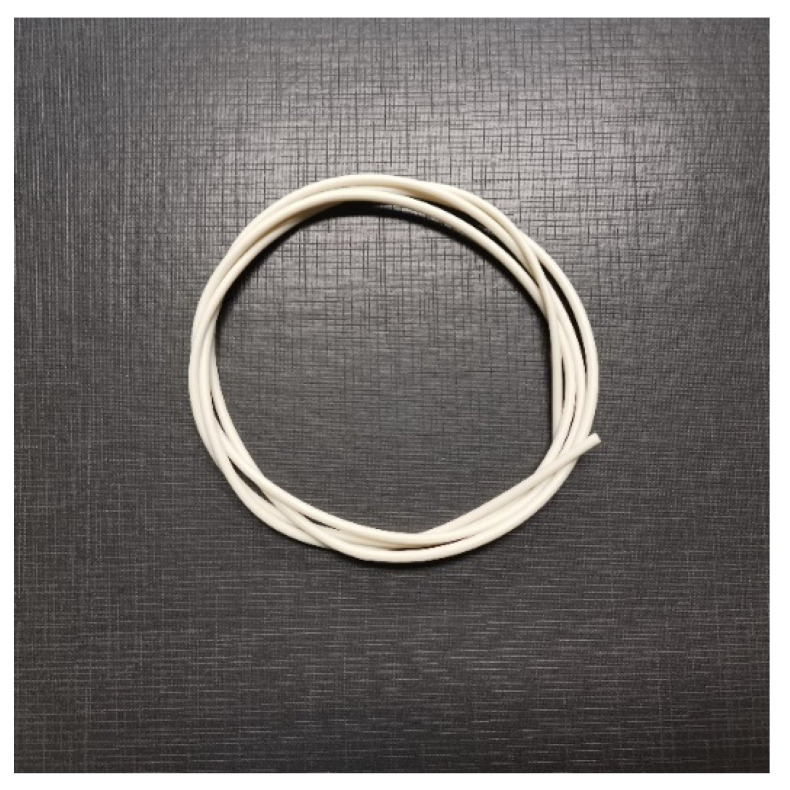
Extruded filament of ENR-50/PCL at 50/50.

After the suitable blend proportion was discovered, the ENR-50/PCL 50/50 *w*/*w*, compound was preliminarily extruded into the filament as shown in Figure 8. The filament was primarily used for thermal and MFI testing to investigate the printing conditions. It was found that the ENR-50/PCL at 50/50 *w*/*w* was able to form neat, smooth, printable filament with a good required dimension of 1.75 mm in diameter. The printing temperature and printing speed along with the melt flow index will be used to optimize the printing conditions and will be presented in future work.

## 4. Conclusions

Important characteristics of a material for use in an FDM machine are the melting temperature, the melt flow rate, and the mechanical properties. The morphology of fractured surfaces was able to support most of the results quite well. The clear fractured surfaces of the blends confirmed weak interactions and poor bonding between rubber and plastic phases. Platelet and droplet domains were found with high contents of PCL (≥50% *w*/*w*). The melting temperatures of both blends were slightly changed from the melting temperature of PCL. This means that PCL strongly affected the melting temperatures of the blends, although the amount of PCL was much less than that of the rubbers. The melt flow rate of the ENR-50/PCL blend was considerably better than the STR5L/PCL blend, and the PCL played a major role. The unvulcanized rubber may have contributed to large permanent deformations of both blends. The hardness decreased with rubber content as it was the naturally softer phase. From tensile testing, a high content of rubber degraded the load capacity, while ENR-50 performed better than STR5L. The ENR-50 blends had higher tensile strengths than the STR5L blends because ENR-50 was more miscible and compatible with PCL than STR5L, creating high continuity of the rigid PCL phase. In contrast to strength, the elongation at break increased with content of rubber. The uniform and good dispersion of ENR-50 in the PCL phase, as well as its properties, confirmed that ENR-50 is potentially a good choice as an elastic and soft material to blend with PCL for FDM.

## Figures and Tables

**Table 1:** Compound formulations.

Sample Code	Sample Name	Quantity (*w*/*w*)			Quantity (phr)		
		STR5L	ENR-50	PCL	ZnO	Wingstay L	Paraffinic Oil
A	STR5L/PCL 40/60	40		60	5	1	20
B	STR5L/PCL 50/50	50		50	5	1	20
C	STR5L/PCL 60/40	60		40	5	1	20
D	ENR-50/PCL 40/60		40	60	5	1	20
E	ENR-50/PCL 50/50		50	50	5	1	20
F	ENR-50/PCL 60/40		60	40	5	1	20

**Table 2:** Mixing method.

Step	Time (min)	Component Adding
1	5	Rubber (STR5L or ENR-50)
2	2	Zinc oxide
3	2	Wingstay L
4	5	Paraffinic oil
5	-	Unloading rubber compound
6	5	PCL
7	5	ENR-50 or STR5L compound

**Table 3:** Glass transition temperatures (T_g_), melting temperature (T_m_), and heat of fusion (∆H) for PCL, A = STR5L/PCL at 40/60, B = STR5L/PCL at 50/50, C = STR5L/PCL at 60/40, D = ENR-50/PCL at 40/60, E = ENR-50/PCL at 50/50, and F = ENR-50/PCL at 60/40.

Samples	T_g1_ (°C)	T_g2_ (°C)	T_m_ (°C)	∆H (J/g)
PCL	−53.47	-	50.9	51.8
A	−72.01	−27.87	50.6	13.6
B	−72.92	-	49.5	11.2
C	−71.72	−39.29	49.0	17.9
D	−29.81	−9.45	49.9	37.6
E	−32.77	−10.27	48.4	18.0
F	−30.55	−14.93	49.1	22.2

T_g_ of STR5L = −70 to −72 °C, T_g_ of ENR-50 = −14 to −18 °C.
